# Predicted antiviral activity of tenofovir versus abacavir in combination with a cytosine analogue and the integrase inhibitor dolutegravir in HIV-1-infected South African patients initiating or failing first-line ART

**DOI:** 10.1093/jac/dky428

**Published:** 2018-10-31

**Authors:** Anne Derache, Collins C Iwuji, Siva Danaviah, Jennifer Giandhari, Anne-Geneviève Marcelin, Vincent Calvez, Tulio de Oliveira, François Dabis, Deenan Pillay, Ravindra K Gupta

**Affiliations:** 1 Africa Health Research Institute (AHRI), KwaZulu-Natal, South Africa; 2 Division of Infection and Immunity, University College London, London, UK; 3 Institute for Global Health, University College London, London, UK; 4 Department of Global Health and Infection, Brighton and Sussex Medical School, Brighton, UK; 5 KwaZulu-Natal Research Innovation and Sequencing Platform (KRISP), School of Laboratory Medicine and Medical Sciences, College of Health Sciences, University of KwaZulu-Natal, Durban, South Africa; 6 Sorbonne Université, INSERM, Institut Pierre Louis d’Epidémiologie et de Santé Publique (iPLESP), AP-HP, Hôpital Pitié-Salpêtrière, Laboratoire de virologie, F-75013 Paris, France; 7 Université de Bordeaux, ISPED, Centre INSERM 1219, Bordeaux, France

## Abstract

**Objectives:**

The WHO recently recommended the use of a new first-line ART containing dolutegravir. We investigated the efficacy of NRTI backbones (tenofovir or abacavir with a cytosine analogue) in low- and middle-income countries where there is significant prior exposure to antiretrovirals and drug resistance to NRTIs.

**Methods:**

Within the treatment-as-prevention study in South Africa, we selected participants with available next-generation sequencing (NGS) data for the HIV-1 *pol* gene at trial entry; they were either ART initiators (*n *=* *1193) or already established on ART (*n *=* *94). NGS of the HIV-1 *pol* gene was carried out using MiSeq technology; reverse transcriptase drug resistance mutations (DRMs) were detected at 5% (DRM^5%^) and 20% (DRM^20%^) for all 1287 participants. Genotypic susceptibility was assessed using the Stanford HIVDB resistance interpretation algorithm.

**Results:**

NRTI DRM^20%^ and DRM^5%^ were detected among 5/1193 (0.4%) and 9/1193 (0.8%) of ART initiators, respectively. There was tenofovir exposure in 73/94 (77.7%) of those established on ART, with full susceptibility to abacavir in 57/94 (60.6%) and 56/94 (59.6%) for DRM^20%^ and DRM^5%^, respectively, while 67/94 (71.3%) and 64/94 (68.1%) were fully susceptible to tenofovir, respectively. The differences between tenofovir and abacavir were not statistically significant at the 20% or 5% variant level (*P *=* *0.16 and 0.29, respectively). NGS detection of variants at the 5% level increased detection of K65R in both naive and treated groups. One of 607 integrase sequences carried a DRM^20%^ (Q148R).

**Conclusions:**

Dolutegravir with a cytosine analogue plus tenofovir or abacavir appears to have similar efficacy in South Africans naive to ART. NGS should be considered in HIV drug resistance surveillance.

## Introduction

Countries such as South Africa are implementing a universal test-and-treat strategy recommended by the WHO, therefore increasing the number of people eligible for ART. This policy may lead to higher levels of acquired drug resistance^1^^,^^2^ and transmitted drug resistance^3^ and compromised ART efficacy in a proportion of patients.^4^^,^^5^ For this reason, a robust, cheap and well tolerated fixed-dose combination (FDC) first-line therapy, with a high genetic barrier, is highly desirable. Although NNRTI-based regimens were efficacious, the low genetic barrier to resistance has resulted in an increasing level of pretreatment drug resistance (PDR) to NNRTIs across low- and middle-income countries (LMICs), compounded by the use of thymidine analogues (TAs) prior to availability of tenofovir-based NRTI backbones.^6^ In response to rising PDR in LMICs, the WHO recommended in 2017 the use of a novel antiretroviral, dolutegravir, a second-generation integrase strand transfer inhibitor (INSTI), in countries with a PDR level >10%.^7^ Cheaper to manufacture, dolutegravir appears to be a good candidate with a high genetic barrier to resistance based on first-line ART studies in northern countries with predominantly subtype B viruses.^8–10^

However, the NRTI backbone needs to be carefully chosen as pretreatment NRTI resistance in ART-naive/prior-ART-exposed individuals may compromise the successful implementation of dolutegravir-based first-line therapy.^11^ Moreover, if its implementation is based on a public health approach, some patients already on efavirenz-based first-line ART may be switched to the new recommended first-line therapy, without prior monitoring of virological suppression status. This raises the very real possibility that patients with virological failure and extensive NRTI resistance mutations^1^ could be switched to dolutegravir-based ART.

So far, only the FDCs of abacavir/lamivudine/dolutegravir^12^ and since April 2018 tenofovir/lamivudine/dolutegravir^8^ have been manufactured and are available. In 2009, Sax *et al*.^13^ observed a shorter time to virological failure and to first adverse event in people starting ART with an abacavir/lamivudine backbone, compared with tenofovir/emtricitabine, in combination with either efavirenz or atazanavir. The FDC of tenofovir/emtricitabine/efavirenz has been available since 2010 and recommended as first-line therapy. Wide-scale use of the same NRTI backbone for previous NNRTI and future INSTI-based regimens has raised concerns regarding activity in those patients with PDR or ongoing viral failure at the time of switch from efavirenz to dolutegravir.

We investigated drug resistance to NRTIs, NNRTIs and INSTIs in the ANRS 12249 treatment-as-prevention (TasP) trial in ART re-initiators/initiators, as well as in people failing their first-line ART enrolled in the trial, in order to understand whether abacavir- or tenofovir-based backbones would be more active.

## Methods

### Study design and setting

The ANRS 12249 TasP trial was a cluster-randomized trial implemented in 22 clusters (2 × 11) in the Hlabisa sub-district in rural KwaZulu-Natal, South Africa, from March 2012 to June 2016. There were ∼1000 individuals ≥16 years residing in each cluster with an HIV prevalence of ∼30%. The full trial protocol has been described previously; participants residing in the intervention clusters were offered ART after HIV diagnosis, regardless of their CD4 counts, whereas participants in control clusters were offered ART according to the prevailing South African guidelines (i.e. CD4 count <350 cells/mm^3^ in March 2012, then <500 cells/mm^3^ from January 2015).^14^

### Study population

Participants attending the trial clinics were either ART naive at entry into the trial or already established on ART prescribed through the Hlabisa HIV treatment and care programme. All individuals were asked to complete study questionnaires and provide plasma samples at their first trial clinic visit. Plasma samples were used for viral load (VL) testing, using the Abbott RealTime HIV-1 m2000rt (Abbott Molecular Inc., Des Plaines, IL, USA), as well as for Sanger drug resistance testing in the Africa Health Research Institute diagnostic laboratory when clinically indicated.

### Next-generation sequencing (NGS)

NGS was used to characterize HIV *pol* from participants’ plasma samples with a VL ≥1000 copies/mL, adapting a protocol that was previously described by Gall *et al*.^15^ Briefly, RNAs were extracted from 1 mL of plasma, using the QIAamp Viral RNA Mini Kit (Qiagen, Hilden, Germany) and were eluted in 60 μL of elution buffer. The near-full HIV genome was amplified with four subtype-C-specific primer pairs, generating four overlapping amplicons of 2.1, 2.3, 2.2 and 3.9 kb. DNA concentrations of amplicons were quantified with the Qubit dsDNA HS Assay Kit (Invitrogen, Carlsbad, CA, USA). Diluted at 0.2 ng/μL each, amplicons were pooled equimolarly and prepared for the library using the Nextera XT DNA Library preparation and the Nextera XT DNA sample preparation index kits (Illumina, San Diego, CA, USA), following the manufacturer’s protocol. The runs comprised a total of 96 samples, including three controls: 1 negative sample, 1 inter-run sample and 1 intra-run sample. If amplification or sequencing failed for the *pol* region of the HIV genome, samples were re-amplified with primers that partially covered the *pol* gene, excluding the integrase, and were sequenced in a 386-sample run, with the same controls as the 96-sample run. The intra-run and inter-run controls were used to assess the reproducibility and accuracy of our method (*n *=* *29). The mean identity of the consensus sequences derived for each duplicate was 99.81% (SD = 0.35%). The mean difference between the SNP frequencies, detected from 0.2% to 100%, was 1.4% (SD = 2.5%); therefore, we set with confidence our detection level of minority variants at 5%.

Read assembly was performed using Geneious 10.0.6 software;^16^ briefly, reads between 100 and 300 bp were selected and those with Phred scores <30 were excluded. The sequences were trimmed up to 10 bp from 5′ and 30 bp at the 3′ end and mapped against a subtype C reference sequence (AF411967) annotated for WHO surveillance of drug resistance mutations (DRMs).^17^ Minority variants between 5% and 20% were included when they were also detected by a BaseSpace application, MiCall.^18^ DRMs in the reverse transcriptase and integrase were detected at 20% (DRM^20%^) and 5% (DRM^5%^) levels of detection.

DRM penalty scores and resistance interpretation were estimated using the Stanford HIV Drug Resistance database (https://hivdb.stanford.edu/) for the following drugs: abacavir, cytosine analogues (lamivudine and emtricitabine), zidovudine, tenofovir, efavirenz/nevirapine and dolutegravir. The mutation scores were classified as follows: 0–9, susceptible; 10–29, low-level resistant; and ≥30, resistant. According to the REGA algorithm V10.0.0, the genotypic susceptibility score (GSS) was calculated for the entire abacavir/lamivudine/dolutegravir or tenofovir/lamivudine/dolutegravir regimens; a GSS score ≥3 and ≥2 represents full susceptibility to the regimen amongst ART initiators and ART-exposed persons, respectively.^19^

### Ethics

The trial was approved by the Biomedical Research Ethics Committee (BFC 104/11) at the University of KwaZulu-Natal and the Medicines Control Council of South Africa (ClinicalTrials.gov: NCT01509508; South African National Clinical Trials Register: DOH-27-0512-3974). All trial participants gave written consent or witnessed thumbprint informed consent prior to undertaking any study procedures.

## Results

### Drug resistance cohort description

Of the 1287 participants with available NGS data for the reverse transcriptase gene, 1193 were classified as ART initiators [their ART status was naive (*n *=* *1054), their ART status was unknown (*n *=* *106) or they were previously exposed to a prevention of mother-to-child HIV transmission (PMTCT) regimen (*n *=* *33)], while 94 participants were currently exposed to ART (Table 1). Integrase NGS data were available for 524 ART initiators and 83 ART-exposed participants.

**Table 1.** Demographic and clinical data of study cohort

	*n*	Previous ART^a^	Female (%)	Age (years), median (IQR)	CD4 count (cells/mm^3^), median (IQR)	VL (copies/mL), median (IQR)	Log_10_ VL, median (IQR)	Time on ART (months), median (IQR)
All participants	1287	NA	70.5	33 (25–44)	427 (247–615)	36961 (9253–153947)	4.6 (4.0–5.2)	NA
ART initiators	1193	NA	71.1	32 (25–45)	439 (262–625)	35020 (9095–151406)	4.5 (4.0–5.2)	NA
ART naive	1054	NA	70.6	33 (25–45)	431 (256–618)	38016 (9370–159908)	4.6 (4.0–5.2)	NA
ART status unknown	106	NA	67.0	32 (25–46)	505 (320–657)	21269 (8165–69843)	4.3 (3.9–4.8)	NA
previously exposed to a PMTCT regimen	33	NA	100	28 (24–34)	539 (399–675)	17210 (4758–101000)	4.2 (3.7–5.0)	NA
ART exposed	94	NA	62.8	34 (28–41)	255 (131–485)	59660 (14166–180933)	4.8 (4.2–5.3)	40.8 (21.7–69.4)
current ART d4T/ZDV	21	no	66.7	39 (32–44)	198 (154–416)	114132 (51488–190124)	5.1 (4.7–5.3)	67.9 (50.0–88.5)
	2	yes	0.0	60 (57–62)	208 (199–218)	135509 (109888–161129)	5.1 (5.0–5.2)	25.5 (19.4–31.6)
current ART TDF	52	no	63.5	31 (27–38)	355 (203–545)	40428 (8560–143717)	4.6 (3.9–5.2)	24.6 (14.1–36.5)
	19	yes	63.2	36 (31–43)	112 (21–189)	77487 (22337–178762)	4.9 (4.3–5.3)	75.4 (69.3–89.7)

d4T, stavudine; ZDV, zidovudine; TDF, tenofovir; NA, not applicable.

a Participants who had a previous ART regimen with a different NRTI backbone compared with their current ART at time of sampling (either currently on a stavudine/zidovudine regimen, but had tenofovir in the past, or currently on a tenofovir regimen, but had stavudine/zidovudine in the past).

Among participants already on ART at trial entry, all were on an NNRTI-based regimen; 23 were on a TA backbone (zidovudine or stavudine) for a median time of 57.3 months (IQR 38.7–85.8), while 71 were on a tenofovir backbone for a median time of 22.4 months (IQR 11.6–37.2). Among people on a tenofovir-based regimen, 19 were previously on a stavudine-based regimen for a median time of 49.0 months (IQR 44.3–67.4).

The participants included in our study had a median age of 33 years (IQR 25–44) and most were female (70.5%). The overall median of CD4 count was 427 cells/mm^3^ IQR (247–615) and was lower among participants already on ART [255 cells/mm^3^ (IQR 131–485)], while the median VL was 4.6 log_10_ copies/mL (IQR 4.0–5.2) and was not different across the different subgroups.

### DRMs

Among ART initiators, 116/1193 (9.7%) had at least one reverse transcriptase inhibitor (RTI) DRM^20%^, while 54/94 (57.4%) participants who were on ART had detectable RTI DRM^20%^. When minority resistant variants were assessed, 152/1193 (12.7%) of ART initiators and 57/94 (60.6%) of ART-exposed participants had RTI DRM^5%^.

**Figure 1.**
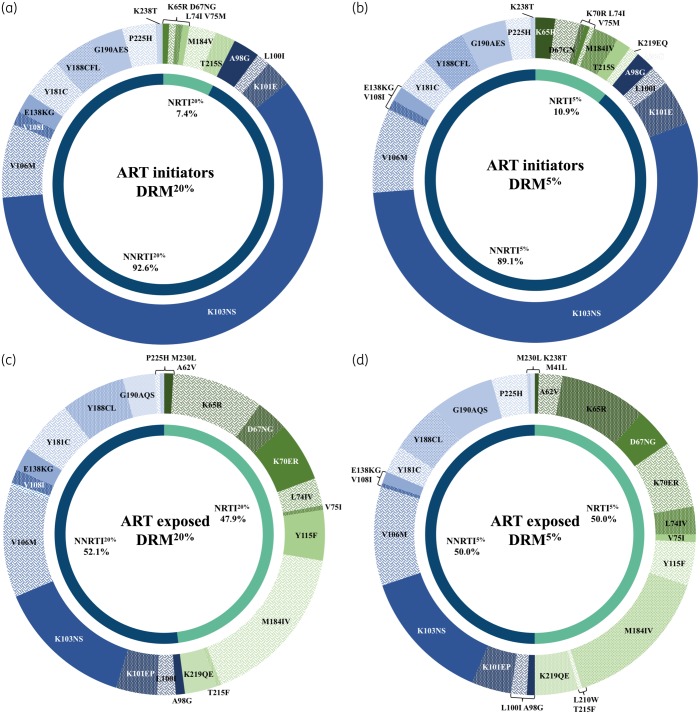
DRMs among ART initiators (a and b) and ART-exposed participants (c and d), at 20% (a and c) and 5% (b and d) levels of detection.

Among all DRM^20%^ detected in ART-initiator participants (*n *=* *148), 137/148 (92.6%) belonged to the NNRTI class, mostly represented by the K103N/S mutations (*n *=* *88/137, 64.2%), while 11/148 (7.4%) were NRTI mutations (Figure 1a). While no major mutation was described for dolutegravir resistance, we found some accessory integrase mutations in 98/524 (18.7%), such as L74I/M (*n *=* *81/98, 82.7%), T97A (*n *=* *8/98, 8.2%) and E157Q (*n *=* *5/98, 5.1%), and more sporadically E138D/K (*n *=* *2), V151I (*n *=* *1) and G163R (*n *=* *1). The main mutations found in the NRTI class were M184V (*n *=* *4/11, 36.4%) associated with cytosine analogues and abacavir resistance, and the main TA mutation (TAM) T215S in its revertant form (*n *=* *3/11, 27.3%). The K65R mutation was found in one participant (*n *=* *1/11, 9.1%). When DRM^5%^ were assessed, double the number of NRTI mutations were found (*n *=* *22), mostly represented by K65R (*n *=* *4/22, 18.2%) and the TAMs D67N and K219EQ (Figure 1b). Nine additional integrase DRM^5%^ were found, mostly L74M (*n *=* *5).

 Among ART-exposed participants, a total of 217 DRM^20%^ were detected and were equally distributed between the NRTI (104/217, 47.9%) and the NNRTI (113/217, 52.1%) classes. Fifteen participants had integrase DRM^20%^ (15/83, 18.1%), including Q148R conferring low-level resistance to dolutegravir, and some accessory integrase mutations were also found: L74I/M (*n *=* *12/15, 80.0%), E157Q (*n *=* *1) and G163R (*n *=* *1). No patients had a major dolutegravir resistance mutation. Among NRTI DRMs, M184IV was the most prevalent (*n *=* *36/104, 34.6%), followed by K65R (20/104, 19.2%) (Figure 1c). K65R was detected in 18/72 (25.0%) and 2/17 (11.8%) participants who were on a tenofovir- and stavudine-based regimen, respectively. TAMs represented 14.4% of the DRMs (*n *=* *15/104); they belonged exclusively to the TAM-2 pathway and were found among nine participants who were previously or currently treated with TAs, with the exception of two participants who initiated ART with a tenofovir-based regimen.

Twenty-two more NRTI DRM^5%^ were detected, mostly represented by the TAM-2 mutations in addition to two mutations from the TAM-1 pathway (M41L and L210W). Notably, two additional K65R mutations were detected at the 5% as compared with the 20% variant threshold (Figure 1d). Three additional participants had integrase DRM^5%^.

### Genotypic antiretroviral susceptibility

**Figure 2.**
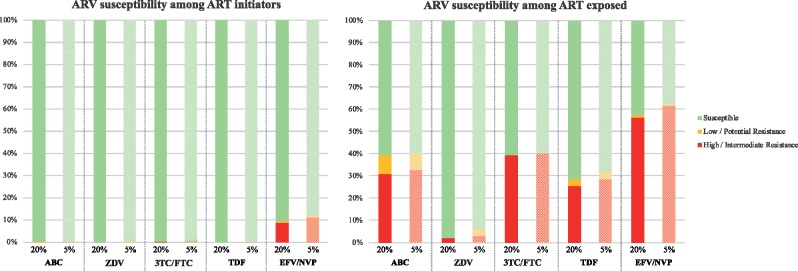
Antiretroviral susceptibility among ART initiators and ART-exposed participants at 20% and 5% variant levels by NGS. ARV, antiretroviral; ABC, abacavir; ZDV, zidovudine; 3TC/FTC, lamivudine/emtricitabine; TDF, tenofovir; EFV/NVP, efavirenz/nevirapine.

When resistance was detected at a 20% variant level, all ART initiators with available consensus sequence covering the entire integrase (*n *=* *524) were fully susceptible to dolutegravir. Only 5 participants out of 1193 (0.4%) had low- to high-level NRTI resistance, while 115 had resistance to NNRTIs (9.6%) (Figure 2). At a variant level of 5%, more participants had RTI resistance detected among ART initiators; 9/1193 participants had NRTI resistance (0.8%), while 145/1193 (12.2%) had resistance to NNRTIs (Figure 2). Whether DRM^20%^ or DRM^5%^ were considered, 99.2% of ART initiators had full susceptibility (GSS ≥3) to both dolutegravir-based regimens. There was no significant difference between the different ART-initiator sub-groups (ART naive, ART status unknown and previously exposed to a PMTCT regimen).

Among ART-exposed participants, only one had low-level resistance to dolutegravir (Q148R) among participants with available consensus sequence covering the integrase (*n *=* *83). This individual had no NRTI mutations. Regarding resistance to NRTI^20%^, 57/94 (60.6%) were fully susceptible to abacavir and cytosine analogues, while 67/94 (71.3%) were fully susceptible to tenofovir (*P *=* *0.16; Figure 2). Accounting for DRM^5%^, 66.0% and 69.1% of participants were predicted to be fully susceptible (GSS ≥2) to abacavir- and tenofovir-based dolutegravir regimens, respectively.

Full susceptibility to zidovudine was found among 92/94 (97.9%). Susceptibility to NNRTIs was compromised for 54/94 (57.4%). When resistance was detected at 5%, full susceptibility to abacavir and cytosine analogues was found for 56/94 (59.6%), while 64/94 (68.1%) were fully susceptible to tenofovir (*P *=* *0.29). Full susceptibility to zidovudine was found among 88/94 (93.6%). Susceptibility to NNRTIs was compromised for 59/94 (62.8%).

## Discussion

In countries hosting large ART programmes with limited treatment options, it is important to evaluate whether the large-scale implementation of a new regimen will be effective. Following recent WHO guidelines,^7^ South Africa will soon roll out a dolutegravir-based regimen as first-line ART. Our study aimed to predict genotypic susceptibility to this new regimen by analysing drug resistance over a large population with diverse ART exposure profiles. We found that a tenofovir + emtricitabine/lamivudine NRTI backbone had similar predicted efficacy to one based on abacavir in place of tenofovir.

We did not find any major mutations associated with high-level dolutegravir resistance irrespective of whether individuals were ART initiators or ART exposed. We did observe Q148R, which is associated with high-level dolutegravir resistance when accompanied by the G140S/A mutations.^20^ Indeed, INSTIs are used only for third-line regimens^21^ and access to them is still limited in most African countries due to their cost.^9^ Using NGS for detecting integrase DRM^5%^ allowed detection of more mutations, which corroborates previous studies in northern countries that have not reported any low-level substitutions or polymorphisms associated with decreased susceptibility to dolutegravir,^22^^,^^23^ or decreased virological response to INSTIs.^24^ However, the choice of the NRTI backbone remains crucial, as NRTI resistance mutations may compromise the full potency of dolutegravir-based regimens. Dolutegravir monotherapy has been shown to be inferior to triple drug therapy with dolutegravir,^25^^,^^26^ though dolutegravir + lamivudine may be effective in high-income settings where resistance testing is done before ART initiation.^27^ Although some studies demonstrated that dolutegravir combined with two NRTIs is still potent despite the presence of NRTI mutations,^28^^,^^29^ it is essential to evaluate which NRTI backbone will be the most appropriate for combination with dolutegravir as first-line ART in the context of large-scale ART programmes, such as in South Africa. Indeed, ongoing virological failure in the absence of effective VL monitoring is associated with increased prevalence of drug resistance^30^ and accumulation of mutations.^31^ Furthermore, subtype-specific differential resistance profiles/propensities must be taken into account, such as the higher prevalence of K65R in subtype C virological failures.^1^^,^^32^^,^^33^

Among ART initiators, including patients who were ART naive, previously exposed to a PMTCT regimen or for whom the ART status could not be clarified, nearly all were fully susceptible to both abacavir and tenofovir, with NRTI resistance found in just under 1% of the population, at both 20% and 5% levels of detection. In this specific population, both abacavir and tenofovir would be fully effective and the level of NNRTI resistance found in our population (>10%) confirmed the need to move towards INSTI-based regimens. Similarly, dolutegravir regimens with either abacavir or tenofovir were predicted to be fully active in 99% of ART-naive participants. Our data suggest that NGS may not provide greater detection of drug resistance over Sanger sequencing under current conditions in ART-naive individuals.

Among patients on ART, or those previously exposed to ART, full susceptibility to abacavir and cytosine analogues was found for 56/94 (59.6%), while 64/94 (68.1%) were fully susceptible to tenofovir using the 5% threshold. When accounting for DRM^5%^, full susceptibility to both dolutegravir regimens was not different and below 70%. Significant additional mutations, including K65R, were observed at the 5% level, but not the 20% level, suggesting NGS might be useful in treatment-experienced patients.

The higher, but non-statistically significant, level of resistance to abacavir compared with tenofovir in the context of use of an FDC containing tenofovir/emtricitabine/efavirenz in this population might be explained by the high prevalence of the M184IV mutations, selected early in regimen failure by cytosine analogues, which can also confer partial resistance to abacavir.^34^ Moreover, the detection of TAMs, likely resulting from prior use of TAs, can enhance the resistance to abacavir in the presence of M184V whilst susceptibility to tenofovir might be increased.^35–38^ We found a low level of resistance to zidovudine among both ART initiators and ART-exposed participants despite the detection of TAMs; this is explained by the fact that most of the TAMs were associated with M184V and/or K65R, mutations known to increase susceptibility to zidovudine.^39^ Therefore, these findings confirm the appropriate use of zidovudine for second-line PI-based therapy, after first-line virological failure. Finally, while the tenofovir-selected mutation K65R confers significant resistance to tenofovir and abacavir,^40^ its combination with M184IV actually increases the level of resistance to abacavir, but decreases resistance to tenofovir.^34^

The preferable choice of using tenofovir as a backbone can be guided by clinical benefits: tenofovir has potent anti-HBV activity,^41^ and given the lack of testing for hepatitis B in most LMICs, where HIV and HBV prevalence is high, tenofovir is the preferred option, over abacavir. Secondly, HLA testing for HLA B*5701 is recommended where abacavir use is being considered due to the possibility of hypersensitivity reactions with this allele.^42^ Although one study found abacavir to be safe in African children regardless of HLA status,^43^ no such study has been conducted in adults. Abacavir use is preferred over tenofovir in children due to bone mineral density considerations,^44^ and therefore based on our data abacavir would be a suitable alternative to tenofovir if similar patterns of HIV drug resistance are found in ART-treated children about to initiate dolutegravir-based ART. In adults, tenofovir is contraindicated in moderate to severe kidney disease and in patients with high fracture risk. In an ageing population these conditions are more likely to occur. Although abacavir has been associated with increased risk of cardiovascular disease in retrospective cohorts,^45^ it is unclear whether this is relevant in black African populations.

This study was limited by a modest number of treated patients relative to naive patients. As expected the vast majority of naive participants were susceptible to both NRTIs and INSTIs. We used predicted activities based on genotypes and rule-based algorithms. Recent data from second-line studies have called into question the utility of such algorithms given better responses to second-line ART in those with more NRTI resistance.^46^ However, in the setting of second-line NRTI, resistance is likely a surrogate of adherence and a boosted PI is likely sufficient to suppress virus in the majority of those with high adherence. Therefore, virological outcome studies are clearly needed to answer the question of impact of resistance to components of dolutegravir-containing ART.

We did not explore integrase resistance outside the integrase gene. Finally, we only genotyped those treated individuals with VL >1000 copies/mL. Although the WHO has defined virological failure as two consecutive VL counts of >1000 copies/mL, it is important to investigate drug resistance and outcomes among patients on ART with VL between 50 and 1000 copies/mL, as these individuals will also likely be switched to new dolutegravir first-line treatment without drug resistance testing.

In conclusion, our data suggest that in LMICs tenofovir and abacavir are predicted to have comparable effectiveness in combination with dolutegravir in treatment-naive individuals.

## References

[1] TenoRes Study Group. Global epidemiology of drug resistance after failure of WHO recommended first-line regimens for adult HIV-1 infection: a multicentre retrospective cohort study. Lancet Infect Dis 2016; 16: 565–75. 26831472 10.1016/S1473-3099(15)00536-8 PMC4835583

[2] Konou AA, Dagnra AY, Vidal N et al Alarming rates of virological failure and drug resistance in patients on long-term antiretroviral treatment in routine HIV clinics in Togo. AIDS 2015; 29: 2527–30. 26558549 10.1097/QAD.0000000000000906

[3] Gupta RK, Gregson J, Parkin N et al HIV-1 drug resistance before initiation or re-initiation of first-line antiretroviral therapy in low-income and middle-income countries: a systematic review and meta-regression analysis. Lancet Infect Dis 2018; 18: 346–55. 29198909 10.1016/S1473-3099(17)30702-8 PMC5835664

[4] Ávila-Ríos S, García-Morales C, Matías-Florentino M et al Pretreatment HIV-drug resistance in Mexico and its impact on the effectiveness of first-line antiretroviral therapy: a nationally representative 2015 WHO survey. Lancet HIV 2016; 3: e579–91. 27658867 10.1016/S2352-3018(16)30119-9

[5] Hamers RL, Schuurman R, Sigaloff KC et al Effect of pretreatment HIV-1 drug resistance on immunological, virological, and drug-resistance outcomes of first-line antiretroviral treatment in sub-Saharan Africa: a multicentre cohort study. Lancet Infect Dis 2012; 12: 307–17. 22036233 10.1016/S1473-3099(11)70255-9

[6] Gregson J, Kaleebu P, Marconi VC et al Occult HIV-1 drug resistance to thymidine analogues following failure of first-line tenofovir combined with a cytosine analogue and nevirapine or efavirenz in sub Saharan Africa: a retrospective multi-centre cohort study. Lancet Infect Dis 2017; 17: 296–304. 27914856 10.1016/S1473-3099(16)30469-8 PMC5421555

[7] WHO. *Transition to New Antiretrovirals in HIV Programmes* 2017 http://apps.who.int/iris/bitstream/handle/10665/255888/WHO-HIV-2017.20-eng.pdf;jsessionid=B8A2CE2FB9211C7981D3670349FBEDDF?sequence=1.

[8] USAID. *The Dolutegravir Opportunity.* 2017 https://www.ghsupplychain.org/sites/default/files/2017-10/HIV-AIDS%20TLD%201%20pager.pdf.

[9] Venter WF, Kaiser B, Pillay Y et al Cutting the cost of South African antiretroviral therapy using newer, safer drugs. S Afr Med J 2016; 107: 28–30. 28112085 10.7196/SAMJ.2016.v107.i1.12058

[10] Walmsley S, Baumgarten A, Berenguer J et al Dolutegravir plus abacavir/lamivudine for the treatment of HIV-1 infection in antiretroviral therapy-naive patients: week 96 and week 144 results from the SINGLE randomized clinical trial. J Acquir Immune Defic Syndr 2015; 70: 515–9. 26262777 10.1097/QAI.0000000000000790 PMC4645960

[11] Rhee SY, Shafer RW. Surveillance Drug Resistance Mutation (SDRM) Worksheet: NRTIs. Stanford University HIV Drug Resistance Database, 2016 https://hivdb.stanford.edu/pages/SDRM.worksheet.NRTI.html.

[12] ViiV Healthcare. *Triumeq*^®^ https://www.viivhealthcare.com/our-medicines/triumeq.aspx.

[13] Sax PE, Tierney C, Collier AC et al Abacavir-lamivudine versus tenofovir-emtricitabine for initial HIV-1 therapy. N Engl J Med 2009; 361: 2230–40. 19952143 10.1056/NEJMoa0906768 PMC2800041

[14] Iwuji CC, Orne-Gliemann J, Larmarange J et al Universal test and treat and the HIV epidemic in rural South Africa: a phase 4, open-label, community cluster randomised trial. Lancet HIV 2018; 5: e116–25. 29199100 10.1016/S2352-3018(17)30205-9

[15] Gall A, Ferns B, Morris C et al Universal amplification, next-generation sequencing, and assembly of HIV-1 genomes. J Clin Microbiol 2012; 50: 3838–44. 22993180 10.1128/JCM.01516-12 PMC3502977

[16] Kearse M, Moir R, Wilson A et al Geneious Basic: an integrated and extendable desktop software platform for the organization and analysis of sequence data. Bioinformatics 2012; 28: 1647–49. 22543367 10.1093/bioinformatics/bts199 PMC3371832

[17] Bennett DE, Camacho RJ, Otelea D et al Drug resistance mutations for surveillance of transmitted HIV-1 drug-resistance: 2009 update. PLoS One 2009; 4: e4724. 19266092 10.1371/journal.pone.0004724 PMC2648874

[18] Lapointe HR, Dong W, Lee GQ et al HIV drug resistance testing by high-multiplex “wide” sequencing on the MiSeq instrument. Antimicrob Agents Chemother 2015; 59: 6824–33. 26282425 10.1128/AAC.01490-15 PMC4604392

[19] Camacho R, Van Laethem K, Geretti AM et al Algorithm for the Use of Genotypic HIV-1 Resistance Data (Version Rega v10.0.0 *)* 2017 https://rega.kuleuven.be/cev/avd/software/rega-hiv1-rules-v10.pdf.

[20] Liu TF, Shafer RW. Web resources for HIV type 1 genotypic-resistance test interpretation. Clin Infect Dis 2006; 42: 1608–18. 16652319 10.1086/503914 PMC2547473

[21] WHO. *Consolidated Guidelines on the Use of Antiretroviral Drugs for Treating and Preventing HIV Infection.* 2016 http://apps.who.int/iris/bitstream/handle/10665/208825/9789241549684_eng.pdf?sequence=1.

[22] Ambrosioni J, Nicolás D, Manzardo C et al Integrase strand-transfer inhibitor polymorphic and accessory resistance substitutions in patients with acute/recent HIV infection. J Antimicrob Chemother 2017; 72: 205–9. 27624569 10.1093/jac/dkw376

[23] Casadellà M, van Ham PM, Noguera-Julian M et al Primary resistance to integrase strand-transfer inhibitors in Europe. J Antimicrob Chemother 2015; 70: 2885–8. 26188038 10.1093/jac/dkv202

[24] Nguyen TT, Bocar Fofana D, Charpentier C et al Prevalence and clinical impact of minority resistant variants to integrase inhibitors In: Abstracts of the Conference on Retroviruses and Opportunistic Infections, Boston, MA, USA, 2018. Abstract 545. Foundation for Retrovirology and Human Health, Alexandria, VA, USA.

[25] Heredia A, Hassounah S, Medina-Moreno S et al Monotherapy with either dolutegravir or raltegravir fails to durably suppress HIV viraemia in humanized mice. J Antimicrob Chemother 2017; 72: 2570–3. 28637235 10.1093/jac/dkx195 PMC5890682

[26] Wijting I, Rokx C, Boucher C et al Dolutegravir as maintenance monotherapy for HIV (DOMONO): a phase 2, randomised non-inferiority trial. Lancet HIV 2017; 4: e547–54. 29107562 10.1016/S2352-3018(17)30152-2

[27] Taiwo BO, Marconi VC, Berzins B et al Dolutegravir plus lamivudine maintains HIV-1 suppression through week 48 in a pilot randomized trial. Clin Infect Dis 2017; 66: 1794–7. 10.1093/cid/cix1131 PMC5961309 29293895

[28] Demarest J, Underwood M, St Clair M et al Dolutegravir-based regimens are active in integrase strand transfer inhibitor-naive patients with nucleoside reverse transcriptase inhibitor resistance. AIDS Res Hum Retroviruses 2014; 34: 343–6. 10.1089/aid.2017.0184 PMC5899294 29444582

[29] Sörstedt E, Carlander C, Flamholc L et al Effect of dolutegravir in combination with nucleoside reverse transcriptase inhibitors (NRTIs) on people living with HIV who have pre-existing NRTI mutations. Int J Antimicrob Agents 2018; 51: 733–8. 29371105 10.1016/j.ijantimicag.2018.01.009

[30] Gupta RK, Hill A, Sawyer AW et al Virological monitoring and resistance to first-line highly active antiretroviral therapy in adults infected with HIV-1 treated under WHO guidelines: a systematic review and meta-analysis. Lancet Infect Dis 2009; 9: 409–17. 19555900 10.1016/S1473-3099(09)70136-7

[31] Goodall RL, Dunn DT, Nkurunziza P et al Rapid accumulation of HIV-1 thymidine analogue mutations and phenotypic impact following prolonged viral failure on zidovudine-based first-line ART in sub-Saharan Africa. J Antimicrob Chemother 2017; 72: 1450–5. 28160504 10.1093/jac/dkw583 PMC5400089

[32] Coutsinos D, Invernizzi CF, Moisi D et al A template-dependent dislocation mechanism potentiates K65R reverse transcriptase mutation development in subtype C variants of HIV-1. PLoS One 2011; 6: e20208. 21655292 10.1371/journal.pone.0020208 PMC3105016

[33] Sunpath H, Wu B, Gordon M et al High rate of K65R for antiretroviral therapy-naive patients with subtype C HIV infection failing a tenofovir-containing first-line regimen. AIDS 2012; 26: 1679–84. 22739389 10.1097/QAD.0b013e328356886d PMC3757561

[34] Whitcomb JM, Parkin NT, Chappey C et al Broad nucleoside reverse-transcriptase inhibitor cross-resistance in human immunodeficiency virus type 1 clinical isolates. J Infect Dis 2003; 188: 992–1000. 14513419 10.1086/378281

[35] Lanier ER, Ait-Khaled M, Scott J et al Antiviral efficacy of abacavir in antiretroviral therapy-experienced adults harbouring HIV-1 with specific patterns of resistance to nucleoside reverse transcriptase inhibitors. Antivir Ther 2004; 9: 37–45. 15040535 10.1177/135965350400900102

[36] Miller MD, Margot N, Lu B et al Genotypic and phenotypic predictors of the magnitude of response to tenofovir disoproxil fumarate treatment in antiretroviral-experienced patients. J Infect Dis 2004; 189: 837–46. 14976601 10.1086/381784

[37] Ross L, Parkin N, Chappey C et al Phenotypic impact of HIV reverse transcriptase M184I/V mutations in combination with single thymidine analog mutations on nucleoside reverse transcriptase inhibitor resistance. AIDS 2004; 18: 1691–6. 15280780 10.1097/01.aids.0000131355.44834.e4

[38] Tisdale M, Alnadaf T, Cousens D. Combination of mutations in human immunodeficiency virus type 1 reverse transcriptase required for resistance to the carbocyclic nucleoside 1592U89. Antimicrob Agents Chemother 1997; 41: 1094–8. 9145875 10.1128/aac.41.5.1094 PMC163856

[39] Ly JK, Margot NA, MacArthur HL et al The balance between NRTI discrimination and excision drives the susceptibility of HIV-1 RT mutants K65R, M184V and K65R+M184V. Antivir Chem Chemother 2007; 18: 307–16. 18320935 10.1177/095632020701800603

[40] Petropoulos CJ, Parkin NT, Limoli KL et al A novel phenotypic drug susceptibility assay for human immunodeficiency virus type 1. Antimicrob Agents Chemother 2000; 44: 920–8. 10722492 10.1128/aac.44.4.920-928.2000 PMC89793

[41] Peters L, Mocroft A, Grint D et al Uptake of tenofovir-based antiretroviral therapy among HIV-HBV-coinfected patients in the EuroSIDA study. Antivir Ther 2018; doi:10.3851/IMP3218. 10.3851/IMP3218 29303483

[42] Tangamornsuksan W, Lohitnavy O, Kongkaew C et al Association of HLA-B*5701 genotypes and abacavir-induced hypersensitivity reaction: a systematic review and meta-analysis. J Pharm Pharm Sci 2015; 18: 68–76. 25877443 10.18433/j39s3t

[43] Jesson J, Dahourou DL, Renaud F et al Adverse events associated with abacavir use in HIV-infected children and adolescents: a systematic review and meta-analysis. Lancet HIV 2016; 3: e64–75. 26847228 10.1016/S2352-3018(15)00225-8

[44] Siberry GK, Jacobson DL, Kalkwarf HJ et al Lower newborn bone mineral content associated with maternal use of tenofovir disoproxil fumarate during pregnancy. Clin Infect Dis 2015; 61: 996–1003. 26060285 10.1093/cid/civ437 PMC4551007

[45] Group DADS, Sabin CA, Worm SW et al Use of nucleoside reverse transcriptase inhibitors and risk of myocardial infarction in HIV-infected patients enrolled in the D:A:D study: a multi-cohort collaboration. Lancet 2008; 371: 1417–26. 18387667 10.1016/S0140-6736(08)60423-7 PMC2688660

[46] Paton NI, Kityo C, Thompson J et al Nucleoside reverse-transcriptase inhibitor cross-resistance and outcomes from second-line antiretroviral therapy in the public health approach: an observational analysis within the randomised, open-label, EARNEST trial. Lancet HIV 2017; 4: e341–8. 28495562 10.1016/S2352-3018(17)30065-6 PMC5555436

